# Analysis of Transcriptome Difference between Blood-Fed and Starved Tropical Bed Bug, *Cimex hemipterus* (F.) (Hemiptera: Cimicidae)

**DOI:** 10.3390/insects13040387

**Published:** 2022-04-14

**Authors:** Li Lim, Abdul Hafiz Ab Majid

**Affiliations:** 1 Household & Structural Urban Entomology Laboratory, Vector Control Research Unit, School of Biological Sciences, Universiti Sains Malaysia, Minden 11800, Malaysia; limli110376.ll@gmail.com; 2 Centre for Insect Systematics (CIS), Faculty of Science and Technology, Universiti Kebangsaan Malaysia (UKM), Bangi 43600, Malaysia

**Keywords:** *Cimex hemipterus*, transcriptome, blood-fed, starvation

## Abstract

**Simple Summary:**

Bed bugs are well known for their extreme resilience to starvation. The molecular mechanisms behind this ability, however, are little known. Thus, the whole transcriptomes of blood-fed and starved bed bugs from the species *Cimex hemipterus* (tropical bed bugs) were sequenced and compared. The transcriptome of tropical bed bugs was initially annotated. Following differentially expressed genes (DEGs) analysis, regulated transcripts were mostly identified in biological processes during blood-feeding and starvation. The results provide an overview of the functional genes proportion of this species and a deeper understanding of the bed bug’s molecular mechanism of resistance to blood feeding and starvation.

**Abstract:**

The reference transcriptome for *Cimex hemipterus* (tropical bed bug) was assembled de novo in this study, and differential expression analysis was conducted between blood-fed and starved tropical bed bug. A total of 24,609 transcripts were assembled, with around 79% of them being annotated against the Eukaryotic Orthologous Groups (KOG) database. The transcriptomic comparison revealed several differentially expressed genes between blood-fed and starved bed bugs, with 38 of them being identifiable. There were 20 and 18 genes significantly upregulated in blood-fed and starved bed bugs, respectively. Differentially expressed genes (DEGs) were revealed to be associated with regulation, metabolism, transport, motility, immune, and stress response; endocytosis; and signal transduction. The Kyoto Encyclopedia of Genes and Genomes (KEGG) pathway enrichment analysis showed an enrichment of genes encoding steroid biosynthesis, glycosaminoglycan biosynthesis, butanoate metabolism, and autophagy in both blood-fed and starved bed bugs. However, in blood-fed bed bugs, genes involved in histidine metabolism, caffeine metabolism, ubiquinone/terpenoid-quinone biosynthesis, and sulfur relay system were enriched. On the other hand, starvation activates genes related to nicotinate and nicotinamide metabolism, fatty acid elongation, terpenoid backbone biosynthesis, metabolism of xenobiotics by cytochrome P450, riboflavin metabolism, apoptosis, and protein export. The present study is the first to report a de novo transcriptomic analysis in *C. hemipterus* and demonstrated differential responses of bed bugs in facing blood-feeding and starvation.

## 1. Introduction

*Cimex hemipterus*, the tropical bed bug, is an obligatory blood-feeding insect [1] and one of the most difficult to control in urban areas. Not only are they well known for their insecticide resistance, but they are also well known for their extreme resilience to starvation and dehydration, allowing them to survive long periods between blood meals [2]. Bed bugs may exhibit adapting mechanisms to this stressful situation, such as thin cuticle with a low water permeability, a slow respiration rate, and clustering to reduce water loss [3,4]. Nevertheless, the molecular mechanism behind this ability is elusive.

Biotechnological approaches have emerged as an alternative to pest management, and advancements in next-generation sequencing (NGS) development simplified the process of assembling a transcriptome and analyzing gene expression patterns in an organism or acquiring genetic information about a target pest [5,6]. Thus, to better understand how tropical bed bugs cope with fluctuating food resources, comparative transcriptome analysis based on Illumina high-throughput sequencing was performed on the blood-fed and starved (45 days post-feeding) tropical bed bugs. The regulatory pathways or genes responsible for surviving a blood meal and prolonged starvation could be revealed, benefitting pest management.

## 2. Materials and Methods

### 2.1. Bed Bugs Samples

The strain of *Cimex hemipterus* used in this study was collected in Kuala Lumpur International Airport (KLIA) in 2014 and maintained at the Household and Structural Urban Entomology Laboratory, Vector Control Research Unit, School of Biological Sciences, Universiti Sains Malaysia, since that time [7]. At 23 ± 1 °C, the bed bugs colony were maintained in plastic containers (200 mL) covered with cloth mesh and fed directly on the volunteer arm once every two weeks. The feeding of bed bugs on human blood was conducted following the designated protocol by the code USM/JEPeM/19120868 and approved by the Human Research Ethics Committee USM (HREC). Newly emerged male adult tropical bed bugs were used in this experiment, and they were allowed to feed on a human volunteer’s arm. Two tested treatments were conducted: tropical bed bugs under blood-fed state (bed bugs were euthanized and preserved in absolute ethanol after feeding) and bed bugs under a starved state (bed bugs were euthanized and preserved in absolute ethanol 45 days post-feeding), each with two biological replications (one individual bed bug per replicate). The samples were coded as FBB1 (sample 1) and FBB2 (sample 2) for blood-fed bed bugs and SBB1 (sample 1) and SBB2 (sample 2) for starved bed bugs.

### 2.2. RNA Isolation, Library Preparation and Sequencing

Each bed bug sample was sterilized with 75% ethanol and then rinsed with sterile distilled water. A sterile micro pestle was used to grind the samples, and total RNA was extracted using a HiYield Total RNA Mini PCR Kit (Real Biotech Corporation, Taipei, Taiwan). After extraction, the concentration and purity (OD260/280 detection) of RNA was assessed by a Nanodrop spectrophotometer (Labgene Scientific, Châtel-saint-denis, Switzerland). Agarose gel electrophoresis was also used to assess the integrity of the RNA samples, and Agilent2100 (Agilent Technologies, Santa Clara, CA, USA) was used for RIN value mensuration. Only RNA samples with a purity of 1.80–2.20 and an RIN ≥ 8.0 were used for library construction.

Qualified RNA samples were processed for cDNA library construction using the Truseq RNA sample prep Kit (Illumina, San Diego, CA, USA) following the manufacturer’s instructions. The samples were then sequenced using the Illumina HiSeq 2500 platform.

The raw data have been submitted to the Sequence Read Archive (SRA) database with accession numbers SAMN18780127 and SAMN18780128 under BioProject, PRJNA640473, while the assembled data are available in the figshare repository (DOI: https://doi.org/10.6084/m9.figshare.18393767.v1, accessed on 10 January 2022).

### 2.3. Initial Sequence Processing and Analysis of Reads

The following workflow was performed on Linux system Ubuntu (64-bit) with 6 CPUs processor and 13,000 MB base memory. The adapter, ploy-N, and low-quality reads were removed from the raw reads using Cutadapt (ver1.9.1) [8]. FastQC was used to estimate the quality of the clean reads obtained [9]. The number of clean paired-end reads with 150 bp were 22,811,975; 20,768,077; 22,030,403; and 21,707,859 for samples FBB1, FBB2, SBB1, and SBB2, respectively. The de novo assembly of high-quality clean raw reads was performed using SOAPdenovo-Trans (ver1.03) with default parameter [10] and gap-filled was performed using GapCloser (ver1.12-r6) [11]. The quality of the assembly was assessed using rnaQUAST (ver1.4.0) [12]. The assembled genes were annotated against the Eukaryotic Orthologous Groups (KOG) databases [13].

The Kallisto program (ver0.46.0) was used to quantify the abundances of transcripts from RNA-Seq data [14]. The counts matrix prepared using Kallisto program was submitted to a web server using Integrative Differential Expression Analysis for Multiple Experiments (IDEAMEX) [15] for differential expression analysis using the DESeq2 method. The thresholds, *p*-value < 0.05, and log2 (fold change) ≥ 1 were set for calculating significantly differential expression of a gene (DEGs). A volcano plot with the *x*-axis, log2 fold changes versus the *y*-axis, and the adjusted *p*-value was generated to visualize the differential expression between samples, with each dot representing a gene.

The Kyoto Encyclopedia of Genes and Genomes (KEGG) [16] enrichment pathway analysis was used to understand the overall expression trends of genes using KOBAS [17], a web server for testing the statistical enrichment of KEGG pathways in bed bugs under blood-fed and starved situations against the *Cimex lectularius* database.

### 2.4. Quantitative Real-Time Polymerase Chain Reaction (qRT-PCR) Validation

qRT-PCR was performed to validate RNA-Seq results. Two genes, Trypsin-1 (PRSS1) and Pickpocket protein 28 (ppk28) were selected from the assembled transcripts as differentially expressed. The 60S ribosomal protein L18 (RPL18) was used as the housekeeping gene [18]. No template control (NTC) was also included in amplification. Primers were designed based on assembled transcriptome sequences (Table 1) using the primer designing tool from NCBI [19]. Apical Scientific SDN. BHD. (Selangor, Malaysia) generated the primer sequences of the selected genes used in qRT-PCR analysis.

qRT-PCR was conducted using SYBR Green qPCR Master Mix, Universal, 2X (Thermo Fisher Scientific, Waltham, MA, USA) in a Rotor-Gene Q real-time PCR system (Qiagen, Düsseldorf, Germany). Two biological replicates were used for each selected gene. The following qRT-PCR procedures were used: initial denaturation, 95 °C for 2 min; followed by 40 cycles of denaturation (95 °C for 5 s) and annealing (65 °C for 15 s). Amplification and melting curves were generated to assess amplification accuracy. Primers that produce a single peak suggest a single amplification product and are selected for qRT-PCR. The cycle threshold (Ct) was determined, and the samples’ relative fold gene expression for each gene was calculated using 2^−∆∆Ct^ (delta-delta Ct method). The mean of the relative fold gene expression was then compared using *t*-test.

## 3. Results

### 3.1. De Novo Assembly of the C. hemipterus Transcriptome

A total of 24,609 transcripts were obtained, with N50 and N75 being 2175 bp and 1208 bp, respectively. The assembly exhibited 38.75% of GC content, and the largest size of the transcript was 39,981,897 bp (Table 2).

### 3.2. Functional Annotation of Unigenes

**Figure 1:**
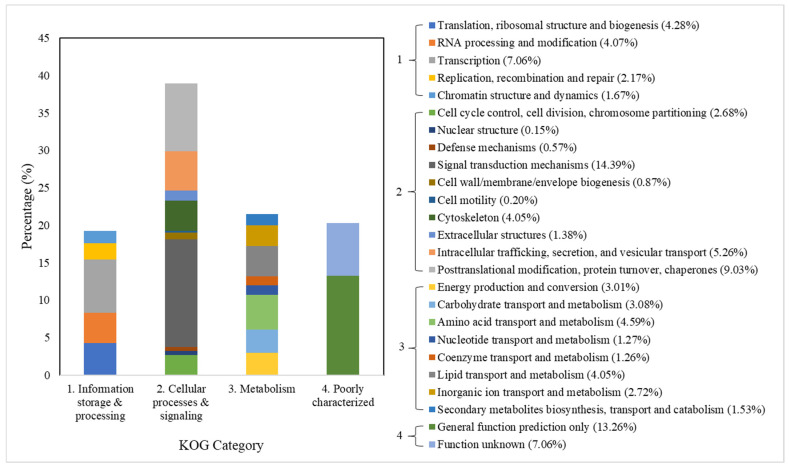
KOG gene function classification of the *C. hemipterus* unigenes.

The unigenes, which refer to a cluster of sequences/genes that perform a particular function, annotated by KOG were classified into four main categories (Figure 1). The highest proportion of unigenes was found in cellular processes and signaling with a value up to 38.58% of the total unigenes, followed by metabolism (21.51), poorly characterized (20.32%), and information storage and processing (19.25%).

### 3.3. Differential Expression Analysis of Blood-Fed and Starved C. hemipterus

**Figure 2:**
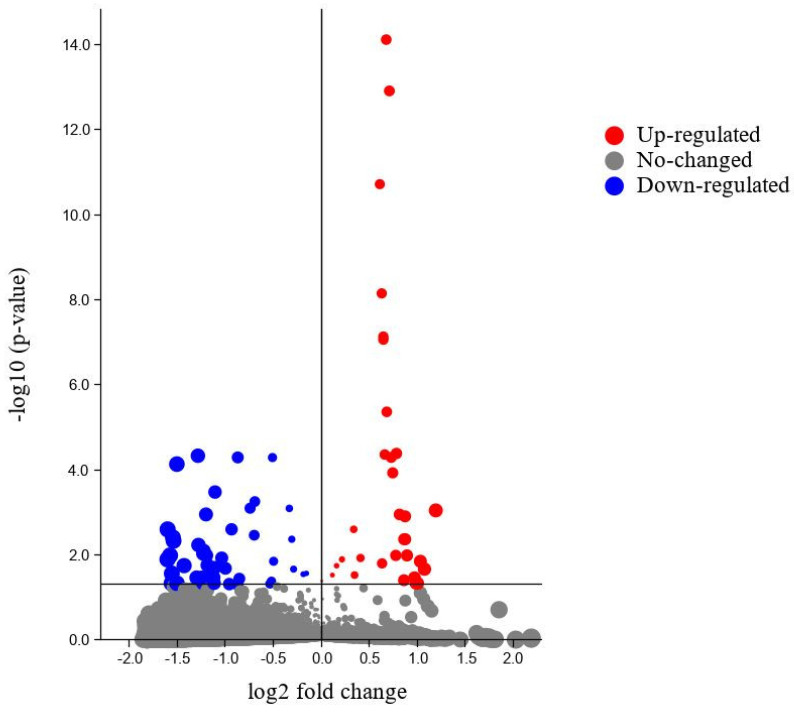
Differentially expressed genes identified in blood-fed and starved bed bugs.

Differential expression analysis showed genes expressed differentially with significant differences in blood-fed and starved bed bugs (Figure 2).

The sequences of the significantly altered genes were blasted against the NCBI and KOG database for identification. The upregulated genes in blood-fed bed bugs could be grouped according to their functions. In general, these genes are associated with oxygen transport (HBA2 and HBB), protein metabolism (EEF1A1, RAB37, coiled-coil protein TPD52, DAN4, and Miga), endocytosis and signal transduction (EHD3, dynein beta chain, GOLGA6L1, transmembrane protein, MFSD14A, and nuclear pore complex), iron metabolism (FTH1 and FTL), locomotion (thymosin beta 4 X-linked), stress response (Hsp70Ba), translation regulation (PKM), and reproduction (MARF1) (Table 3).

On the other hand, upregulated genes in starved bed bugs included those that related to age (ACP7 and PCP36), protein metabolism (FBXL16, E3 ubiquitin ligase, ankyrin repeat protein, and UDP-N-acetylglucosamine--peptide N-acetylglucosaminyltransferase 110 kDa subunit), immunity (CYP, PRG4, ALPK1), excretion (ABCC, GMPR), energy production (TER94 and SBK1), mRNA processing (SNRNP70), locomotion (twk-18), smell perception (general odorant-binding protein), apoptosis (FGFR3), and pyrimidine biosynthesis (PYRE-F) (Table 3).

### 3.4. KEGG Pathway Enrichment Analysis

Table 4 summarizes the KEGG pathway enriched in blood-fed bed bugs, with most pathways related to metabolisms, including histidine metabolism, steroid biosynthesis, caffeine metabolism, ubiquinone/terpenoid-quinone biosynthesis, butanoate metabolism, and glycosaminoglycan biosynthesis.

In starved bed bugs, enriched pathways were also related mainly to metabolisms, including steroid, glycosaminoglycan, butanoate, nicotinate/nicotinamide, fatty acid, riboflavin, terpenoid, and the metabolism of xenobiotics by cytochrome P450. Other pathways could be categorized as cellular processes (Apoptosis, Autophagy) and genetic information processing (Protein export) (Table 4).

Nevertheless, pathways including oxidative phosphorylation, glycosaminoglycan biosynthesis, steroid biosynthesis, and butanoate metabolism were found in both blood-fed and starved bed bugs (Table 4).

### 3.5. qRT-PCR Validation

**Figure 3:**
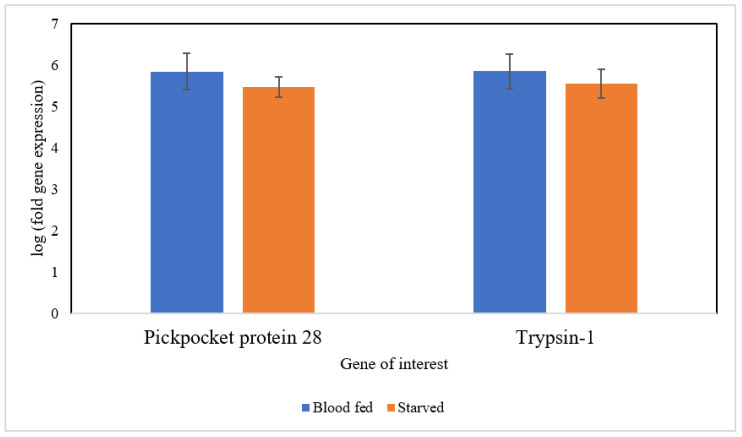
qRT-PCR analysis of two genes: Pickpocket protein 28 and Trypsin-1.

Based on Figure 3, two selected genes, Pickpocket protein 28 (Ppk28), and Trypsin-1 were expressed similarly in blood-fed and starved bed bugs (*p*-value = 0.538 for gene Ppk28 and 0.630 for gene Trypsin-1). The qPCR results showed a similar regulated trend in the DEGs analysis of these genes.

## 4. Discussion

This study is the first to report on the transcriptomic analysis of *C. hemipterus*. The annotation of the assembly has elucidated the fundamental molecular knowledge of *C. hemipterus*, and this transcript information may aid in further molecular research on *C. hemipterus*.

Notably, RNA was collected from blood-fed and starved tropical bed bugs for determining the functional profile of the bed bugs concerning blood meals. Differential expression analysis was used to identify upregulated genes in blood-fed and starved bed bugs. Although most of the transcriptome remains unaltered (Figure 2), a molecular shift indeed occurred in bed bugs following blood meal consumption, with 38 transcripts being differentially expressed.

Differentially expressed genes (DEGs) revealed that the expression of heat shock 70 kDa protein Ba was upregulated. The upregulation is probably due to the thermal stress caused by the intake of large amounts of warm blood, as this protein responds to heat and the immediate increase in its expression to protect the bed bugs from the thermal stress while also activating the signaling pathways in blood processing and digesting [20,21].

After blood feeding, genes related mainly to transport and endocytosis were upregulated. For instance, three genes were upregulated, including hemoglobin subunit alpha 2, hemoglobin subunit beta, and O-acyltransferase-like protein involved in oxygen transport, indicating the bed bugs require higher oxygen and ions transport after blood feeding. The genes encoding the proteins, including dynein beta chain, transmembrane protein, hippocampus abundant transcript 1 protein, and nuclear pore complex are all required for transportation, whether food or oxygen. They either function as a gate to control the macromolecules passage or are involved in cilium biogenesis, which is essential for moving substances. Both EH domain-containing protein 3 and golgin subfamily A member 6-like protein 1 were also upregulated, which is essential for endocytosis, a process to take in substances such as nutrients. These upregulated genes are essential for nutrient absorption and distribution and correspond to bed bugs’ states following blood feeding.

Transcripts involved in protein transport and syntheses such as eukaryotic translation elongation factor 1 alpha 1, ras-related protein Rab-37, coiled-coil protein TPD52, mitoguardin, and cell wall protein DAN4 were also upregulated. The upregulation is reasonable given that the primary composite of blood includes proteins (such as albumin, clotting factors, antibodies, enzymes, and hormones) [22]. Increased amino acid catabolism may result in molecules involved in gluconeogenesis and subsequently into the tricarboxylic acid cycle (TCA) cycle [23]. Iron levels found in hemoglobin should be elevated in blood-fed bed bugs during blood meal processing. Both heavy and light ferritin chains were upregulated, probably to deal with the increased iron level through siderophore uptake and oxidative stress management [24].

Since blood meal is essential for bed bug’s developmental progression and mating [2], there was also an increase in the related genes following bed bug’s blood feeding. For example, thymosin beta 4 X-linked gene expression is essential for the organization of the cytoskeleton. At the same time, pyruvate kinase and meiosis regulator and mRNA stability factor 1 are proteins that are essential in meiosis.

Many species are commonly suffered intermittent food shortages. Thus, the starvation of insects could trigger a response by increasing the storage of resources (energy or water) utilized during starvation or by lowering metabolic rates [25,26]. This study discovered that starvation significantly alters some of the functional transcript such as genes involved in proteolysis. In contrast to blood-fed bed bugs, whose protein metabolism is mainly degraded and synthesized, the protein metabolism in starved bed bugs is likely dominated by proteolysis. The genes encoding F-box/LRR-repeat protein 16, E3 ubiquitin ligase, ankyrin repeat protein, UDP-N-acetylglucosamine–peptide N-acetylglucosaminyltransferase 110 kDa subunit are all critical in the UbI conjugation pathway, which participates in protein processing, activation, conjugation or deconjugation, and signaling proteolysis [27]. Nevertheless, further studies are needed to confirm the actual functions of these proteins in bed bugs.

Starvation is a crisis faced by bed bugs. Fibroblast growth factor receptor 3 involved in apoptosis, which produces phagocytic cells capable of engulfing and rapidly removing nearly damaged cells, confers advantages for bed bugs’ life cycle. The same applies to the the upregulation of uridine 5′-monophosphate synthase, which is essential for pyrimidine biosynthesis, enhancing the mode of regulation and flexibility of a metabolic pathway by demonstrating compartmentation within different parts of the cell—cytosol, mitochondria, and chloroplasts, acting as a pacemaker for cell growth and proliferation [28]. Moreover, U1 small nuclear ribonucleoprotein 70 kDa involved in mRNA processing is essential for cell processes. Genes encoding immune-related proteins system are also upregulated, including cytochrome P450, proteoglycan 4, and alpha-protein kinase 1. These genes participate in immune regulation and the repair of damage, thereby enhancing insects’ resistance to starvation [29]. Cytochrome P450s are a group of widespread metabolic enzymes involved in the oxidation of various of endogenous such as steroids and hormones and exogenous compounds, including alcohol, drugs, and environmental pollutants [30]. In this study, starvation upregulates cytochrome P450s, suggesting a critical role for cytochrome P450s in starvation resistance.

Simultaneously, the genes associated with olfactory (general odorant-binding protein) and locomotion (TWiK family of potassium channels protein 18) were also upregulated in starved bed bugs. These are important for the insect to locate a host for a blood meal. The multidrug resistance-associated protein, which mediates the efflux of drugs and their metabolites from cells, and GMP reductase 1, which is essential for Purine metabolism, were also upregulated. These proteins are considered to play a role in excretion or waste removal.

As the bed bugs were left for 45 days after blood feeding, some genes were upregulated, probably due to their “old” age, as they are all considered as developmental proteins. These upregulated proteins such as acid phosphatase type 7 are involved in acid phosphatase activity for gametes maturation [31]. The pupal cuticle protein 36 is a structural constituent of the cuticle, and transitional endoplasmic reticulum ATPase TER94, which is a developmental protein, and serine/threonine-protein kinase SBK1 may be related in brain development control.

Generally, the metabolic rates should be decreased as the duration of starvation increases, and insects that are starved should consume carbohydrates first and reserve lipid for later consumption due to the need for constant energy expenses [25,32]. However, in this study, the KEGG enrichment pathway analysis revealed no changes in lipid, carbohydrate, and energy metabolism following prolonged starvation (Table 4). In both blood-fed and starved bed bugs, oxidative phosphorylation (energy metabolism), glycosaminoglycan biosynthesis (glycan biosynthesis and metabolism), steroid biosynthesis (lipid metabolism), and butanoate metabolism (carbohydrate metabolism) were enriched. Similarly, starving *Gryllus* crickets did not reduce metabolic rates during prolonged starvation [33]. This suggests that a balance or homeostasis between these different types of metabolism may be achieved, which may be a critical role in starvation resistance. Similarly, autophagy should increase in response to starvation as it is a pathway for the cell to redistribute valuable nutrients by digesting their cellular contents via lysosomal machinery and acting a protective mechanism by helping in the reduction in apoptosis in fat cells, which is essential for the insect maintenance [5,34,35]. Nevertheless, the autophagy pathway was identified in both blood-fed and starved bed bugs.

There was an increase in KEGG enrichments following blood feeding, indicating alterations in biological processes including histidine metabolism, sulfur relay system, synthesis, and the degradation of ketone bodies, caffeine metabolism, and ubiquinone/terpenoid-quinone biosynthesis (Table 4). Histidine is an essential amino acid that bed bugs could obtain from their blood meal because hemoglobin contains high concentration of histidine [36]. As a result, the blood-fed bed bugs have an enriched histidine metabolism. By playing a role in ATP production, histidine could be used as the energy source to maintain or restore cell viability after starvation [37]. Moreover, histidine has increased iron absorption and exhibits antioxidant capabilities [38]. Moreover, the enriched pathway–sulfur relay system is required for protein folding, sorting, and degradation. Other pathways were also identified after blood feeding, including the synthesis and degradation of ketone bodies, caffeine metabolism, and ubiquinone/terpenoid-quinone biosynthesis, which are involved in other secondary metabolites biosynthesis, and the metabolism of cofactors and vitamins.

Hunger-induced stress may invoke a series of regulated anti-starvation mechanisms. In this study, some pathways, including fatty acid elongation, nicotinate/nicotinamide metabolism, riboflavin metabolism, terpenoid backbone biosynthesis, protein export, ribosome, the metabolism of xenobiotics by cytochrome P450, and drug metabolism—cytochrome P450—were only identified in starved bed bugs (Table 4). With the exception for apoptosis, a typical process of cell growth and death, generally, the identified pathways in starved bed bugs could be divided into two functions: metabolism and the immune system.

In most organisms, fatty acids serve as energy storage and structural components in biomembranes. However, fatty acids and the products of their metabolism, principally hydrocarbons and wax esters, also contribute significantly to the thin layer of cuticular lipids that protect insects from desiccation [39]. Terpenoid is the precursor in producing steroids and sterols. Thus, the constant actives of the terpenoid backbone biosynthesis pathway are important in the regulation of steroids as they regulate a variety of developmental (molting, metamorphosis, and diapause) and biological processes (aging, germline development, and innate immunity) in insects [40]. Riboflavin, nicotinate, and nicotinamide metabolisms remain active in starved bed bugs because vitamin B is produced by the *Wolbachia* endosymbiont and is not obtained from their diet [41,42]. The pathway for ribosomal proteins was also identified in starved bed bugs. Apart from protein biosynthesis, ribosomal proteins have participated in various cellular processes. Thus, in conjunction with protein exports, the pathway for ribosomal proteins is important in insects’ development progression [43]. Similarly to DEGs results, cytochromes P450 are known to metabolize most drugs or xenobiotic substrates using a limited number of enzymes by converting them from a toxic insoluble form to a water-soluble form, playing a vital role in the defense system of starved bed bugs [5,44,45,46].

Pickpocket protein 28 and Trypsin-1 were selected for qRT-PCR validation as these genes were expected to be upregulated in blood-fed and starved bed bugs, respectively. Nevertheless, the expression of these two genes remains similar in bed bugs under different physiological states. DEGs analysis revealed no difference in the expression of these two genes in both situations, and the qRT-PCR results were consistent with the DEGs results. Trypsin-1 is mainly involved in digestion and proteolysis. Thus, the expression of Trypsin-1 after blood-feeding is sensible. The expression of Trypsin-1 remained nearly constant in starved bed bugs, consistent with DEGs analysis, as most genes upregulated in starved bed bugs involved in protein metabolism are involved in proteolysis. Pickpocket protein 28 (Ppk28) acts as an osmosensitive ion channel that mediates the cellular and behavioral response to water. As bed bugs survive only on water obtained from their last blood meal [2], the constitutive expression of Ppk28 may aid in water retention, allowing bed bugs to resist dehydration.

## 5. Conclusions

In general, a transcriptome was generated for *C. hemipterus* to provide a reference for future RNA-Seq-based experiments. The present study also identified genes differentially expressed between the blood-fed and starved bed bugs using DEGs analysis. Enriched pathways were identified between the two physiological states of bed bugs, which are primarily associated with the metabolism. The present data provided insights on the molecular mechanism of bed bugs in facing blood-feeding and how they may overcome the challenges associated with prolonged starvation. It also contributes to advancing knowledge advancement about transcripts in tropical bed bugs, which may serve as a source of target genes for pest management.

## Figures and Tables

**Table 1:** Primer sets of qRT-PCR used in the study.

Target Gene Name	Primer Sequence (5′–3′)	Description
RPL18 (housekeeping gene)	ATCGGCCTCCTATCTCTCTATC	60S ribosomal protein L18
	TACGCAAGGCACAGATCTTC	
PRSS1	ATGTATGAAGGCACGGTTGTCAC	Trypsin-1
	CAGTTACGGCCAAGTTGATTTCG	
ppk28	TCAACAGGCTCAAACGAGAGC	Pickpocket protein 28
	GGGTTCACTTGACTCGGGAAAG	

**Table 2:** Statistics of RNA-seq assembly.

Number of contigs	24,609
Largest contig (bp)	22,971
Total length (bp)	39,981,897
N50	2175
N75	1208
GC%	38.75

**Table 3:** DEGs in blood fed and starved tropical bed bugs.

DE	Gene	Description	log_2_ Fold Change	*p*-Value	Function
Upregulated in blood-fed and downregulated in starved bed bugs	HBA2	Hemoglobin subunit alpha 2	14.29	7.5295 × 10^−15^	Heme binding
	HBB	Hemoglobin subunit beta	13.97	1.2125 × 10^−13^	Heme binding
	Oacyl	O-acyltransferase-like protein	2.95	0.0008	Acyltransferase activity
	EEF1A1	Translation elongation factor EF-1 alpha/Tu	9.69	0.00004	Deliver aminoacyl tRNAs to the ribosome
	RAB37	ras-related protein Rab-37	8.51	0.0011	Protein transport
	N/A	Coiled-coil protein TPD52	1.55	0.0039	Protein homodimerization activity
	DAN4	Cell wall protein DAN4	5.57	0.0119	Anchored component of membrane
	Miga	Mitoguardin	1.63	0.0342	Mitochondrial fusion, protein heterodimerization and homodimerization activities
	EHD3	EH domain-containing protein 3	3.10	0.0006	Cilium biogenesis/degradation, protein transport
	N/A	Dyneins, heavy chain	4.84	0.0128	Cilium biogenesis/degradation
	GOLGA6L1	Golgin subfamily A member 6-like protein 1			Have roles in membrane traffic and Golgi structure
	N/A	Transmembrane protein	3.91	0.0008	Transport
	MFSD14A	Hippocampus abundant transcript 1 protein	3.53	0.0273	Transport
	N/A	Nuclear pore complex	3.43	0.0285	Protein transport
	FTH1	Ferritin heavy chain 1	7.88	0.0103	Iron storage
	FTL	Ferritin light chain	7.53	0.0345	Iron storage
	N/A	Thymosin beta 4 X-linked	8.22	0.0043	Actin monomer binding
	Hsp70Ba	Major heat shock 70 kDa protein Ba	4.53	0.0180	Stress response
	PKM	Pyruvate kinase	4.22	0.0299	Translation regulation
	MARF1	Meiosis regulator and mRNA stability factor 1	2.11	0.0363	Meiosis regulator and mRNA stability factor 1
Upregulated in starved and downregulated in blood fed bed bugs	ACP7	Acid phosphatase type 7	−3.87	5.1334 × 10^−15^	Acid phosphatase activity
	PCP36	Pupal cuticle protein 36	−2.10	0.0011	Structural constituent of cuticle
	FBXL16	F-box/LRR-repeat protein 16	−1.85	0.0081	Ubl conjugation pathway
	N/A	E3 ubiquitin ligase interacting with arginine methyltransferase	−1.82	0.0093	Ubiquitin-mediated protein degradation
	N/A	Ankyrin repeat protein	−1.78	0.0269	Mediate protein-protein interactions
	N/A	UDP-N-acetylglucosamine--peptide N-acetylglucosaminyltransferase 110 kDa subunit	−1.77	0.0341	Ubl conjugation pathway
	CYP	Cytochrome P450	−1.37	0.0128	Detoxification
	PRG4	Proteoglycan 4	−1.79	0.0175	Immune response
	ALPK1	Alpha-protein kinase 1	−1.86	0.0200	Innate immunity
	ABCC	Multidrug resistance-associated protein	−8.86	0.00004	Lipid transport
	GMPR	GMP reductase 1	−1.33	0.0275	Purine metabolism
	TER94	Transitional endoplasmic reticulum ATPase TER94	−3.63	0.0043	ATP hydrolysis activity
	SBK1	Serine/threonine-protein kinase SBK1	−1.84	0.0103	ATP binding
	SNRNP70	U1 small nuclear ribonucleoprotein 70 kDa	−2.43	0.0025	mRNA processing
	Twk-18	TWiK family of potassium channels protein 18	−1.81	0.0059	Locomotion
	N/A	General odorant-binding protein	−3.02	0.00005	Sensory perception of smell
	FGFR3	Fibroblast growth factor receptor 3	−1.56	0.0345	Apoptosis
	PYRE-F	Uridine 5′-monophosphate synthase	−2.64	0.0418	Pyrimidine biosynthesis

**Table 4:** KEGG enriched pathways in blood-fed and starved tropical bed bugs.

Status	Pathway
Blood-fed	Histidine metabolism, sulfur relay system, steroid biosynthesis, synthesis and degradation of ketone bodies, caffeine metabolism, ubiquinone and other terpenoid-quinone biosynthesis, butanoate metabolism, autophagy, glycosaminoglycan biosynthesis—chondroitin sulfate/dermatan sulfate
Starved	Steroid biosynthesis, glycosaminoglycan biosynthesis—chondroitin sulfate/dermatan sulfate, butanoate metabolism, apoptosis, nicotinate and nicotinamide metabolism, fatty acid elongation, terpenoid backbone biosynthesis, autophagy, protein export, metabolism of xenobiotics by cytochrome P450, drug metabolism—cytochrome P450, riboflavin metabolism

## Data Availability

The raw data have been submitted to the Sequence Read Archive (SRA) database with accession numbers SAMN18780127 and SAMN18780128 under BioProject, PRJNA640473, while the assembled data are available in the figshare repository (DOI: https://doi.org/10.6084/m9.figshare.18393767.v1).
